# Extracellular Ca^2+^ Is Required for Fertilization in the African Clawed Frog, *Xenopus laevis*

**DOI:** 10.1371/journal.pone.0170405

**Published:** 2017-01-23

**Authors:** Katherine L. Wozniak, Brianna L. Mayfield, Alexis M. Duray, Maiwase Tembo, David O. Beleny, Marc A. Napolitano, Monica L. Sauer, Bennett W. Wisner, Anne E. Carlson

**Affiliations:** Department of Biological Sciences, University of Pittsburgh, Pittsburgh, Pennsylvania, United States of America; University of Colorado Boulder, UNITED STATES

## Abstract

**Background:**

The necessity of extracellular Ca^2+^ for fertilization and early embryonic development in the African clawed frog, *Xenopus laevis*, is controversial. Ca^2+^ entry into *X*. *laevis* sperm is reportedly required for the acrosome reaction, yet fertilization and embryonic development have been documented to occur in high concentrations of the Ca^2+^ chelator BAPTA. Here we sought to resolve this controversy.

**Methodology/principal finding:**

Using the appearance of cleavage furrows as an indicator of embryonic development, we found that *X*. *laevis* eggs inseminated in a solution lacking added divalent cations developed normally. By contrast, eggs inseminated in millimolar concentrations of BAPTA or EGTA failed to develop. Transferring embryos to varying solutions after sperm addition, we found that extracellular Ca^2+^ is specifically required for events occurring within the first 30 minutes after sperm addition, but not after. We found that the fluorescently stained sperm were not able to penetrate the envelope of eggs inseminated in high BAPTA, whereas several had penetrated the vitelline envelope of eggs inseminated without a Ca^2+^ chelator, or with BAPTA and saturating CaCl_2_. Together these results indicate that fertilization does not occur in high concentrations of Ca^2+^ chelators. Finally, we found that the jelly coat includes >5 mM of readily diffusible Ca^2+^.

**Conclusions/Significance:**

Taken together, these data are consistent with requirement of extracellular Ca^2+^ for fertilization. Based on our findings, we hypothesize that the jelly coat surrounding the egg acts as a reserve of readily available Ca^2+^ ions to foster fertilization in changing extracellular milieu.

## Introduction

Fertilization represents the union of two distinct cell types: the sperm and the egg. Although a substantial amount of work on various species has already been done, many of the signaling pathways and molecular events required for fertilization are unknown. Ca^2+^ is an example of an incompletely understood signaling molecule in fertilization and early embryonic development. Fertilization evokes increased intracellular Ca^2+^ in the zygote, and this Ca^2+^ is essential for egg activation and the earliest events of embryonic development in all sexually reproducing species studied thus far [1, 2]. Moreover, intracellular Ca^2+^ regulates various cellular events in both gametes [3–7]. By contrast, the importance of extracellular Ca^2+^ during fertilization is controversial. For example, entry of extracellular Ca^2+^ into sperm signals processes required for fertilization such as the acrosome reaction [7], and extracellular Ca^2+^ is required for robust motility in *X*. *laevis* sperm [8]. However, fertilization and embryonic development reportedly progress normally in the absence of extracellular Ca^2+^ [9]. Moreover, it is a widely held belief in the field of reproductive physiology that Ca^2+^ is unessential for fertilization in *X*. *laevis* [10–13].

Here we report that extracellular Ca^2+^ is required for fertilization and normal embryonic development in *X*. *laevis* eggs. The Ca^2+^ chelators BAPTA (1,2-Bis(2-Aminophenoxy)ethane-*N*,*N*,*N′*,*N′*-tetraacetic acid) and EGTA (ethylene glycol-bis(β-aminoethyl ether)-N,N,N',N'-tetraacetic acid) inhibited embryonic development in a concentration-response manner, with almost no embryonic development evident in eggs inseminated in chelator concentrations of 3 mM or higher. Incubation of eggs and zygotes in BAPTA at various times before or after insemination revealed that extracellular Ca^2+^ is necessary within the first 30 minutes of *X*. *laevis* insemination. We also found that BAPTA-treated sperm do not penetrate the eggs’ vitelline envelope, consistent with the requirement of extracellular Ca^2+^ for *X*. *laevis* sperm to undergo the acrosome reaction [7]. Finally, we found that the jelly coat surrounding the egg includes >5 mM of diffusible Ca^2+^. Taken together, these results indicate that extracellular Ca^2+^ is crucial for fertilization in *X*. *laevis* and that the jelly coat serves as a readily available source of diffusible Ca^2+^.

## Materials and Methods

### Materials

All chemicals, unless noted, were purchased from Thermo Fisher Scientific (Waltham, MA).

### Solutions

BAPTA (Sigma Aldrich, St. Louis MO) and EGTA were prepared as 10 mM or 50 mM stock solutions (adjusted to pH of 7.8 with NaOH) in deionized water and stored at 4°C until use.

All solutions were made in reverse osmosis treated and Elga purified water (Elga LabWater, Chicago IL). Most gamete preparations and experiments were performed using a version of filter sterilized Modified Ringer’s (MR) solution (100 mM NaCl, 1.6 mM KCl, 2 mM CaCl_2_, 1 mM MgCl_2_, and 5 mM HEPES, pH 7.8) [14]. Experiments performed in divalent-free MR (DVF) lacked added CaCl_2_ and MgCl_2_. Fertilization solutions were prepared by diluting either DVF or MR with purified water to the desired concentration, either 33% MR (MR/3) or 33% DVF (DVF/3) [14], unless otherwise stated. The final pH of all BAPTA and EGTA containing solutions was 7.8.

### Collection of gametes

All animal protocols were conducted using accepted standards of humane animal care, approved by the Animal Care and Use Committee at the University of Pittsburgh. Adult *X*. *laevis* frogs were obtained commercially (NASCO, Fort Atkinson WI) and housed at 18°C on a 12 hour/12 hour light/dark cycle. Ovulation was induced in sexually mature *X*. *laevis* females with injection of 1000 IU of human chorionic gonadotropin (hCG) into the dorsal lymph sac. Following hCG injection, individuals were housed overnight for 14–16 hours at 14°C. Females began ovulating 0–2 hours after moving to room temperature. Eggs were collected on dry plastic petri dishes and were used within 10 minutes of collection.

Testes were collected from sexually mature *X*. *laevis* males that were euthanized by immersion in 3.6 g/L tricaine pH 7.4, for 30 minutes. Dissected testes were stored at 4°C in MR for use the day of dissection or in L-15 media (Gibco 11415–064) for use up to 5 days later. To create a sperm suspension, 1/10 of a testis was minced in DVF. Eggs were inseminated by pipetting the sperm into the petri dish above the egg; the volume of added sperm solution never exceeded 1% of the total fertilization solution.

### Developmental assays

Approximately 15–30 eggs per treatment were inseminated in DVF/3 with or without Ca^2+^ or chelators and then assessed for development. Development was assessed based on the appearance of cleavage furrows, which were typically apparent approximately 90 minutes after sperm addition. Inseminated eggs that did not develop cleavage furrows were scored as undivided. Cleaved embryos were scored as developed at the two-, four-, and eight-cell stages. Each experiment was repeated at least three times, and the depicted error bars represent the standard error of the mean (SEM). Data were analyzed with Igor (WaveMetrics, Lake Oswego, OR) and statistical analyses were performed in Excel (Microsoft).

Concentration-response relationships were calculated by plotting the averaged percentage of developed embryos per experiment versus added concentrations of chelator or CaCl_2_. These plots were then fit with a sigmoidal function (equation 1):

$$Y(x)=Y_{0}+\left(\frac{Y_{\infty }-Y_{0}}{1+e^{\left(\frac{x_{half}-x}{rate}\right)}}\right)$$

where *Y*_*0*_ represents the minimum response, *Y*_*∞*_ is the maximum response, *x*_*half*_ represents the half maximal response, and the *rate* is the slope of the curve.

### Transfer assays

Transfer assays were performed at various time points relative to sperm addition. For these assays, eggs were inseminated in one solution, washed twice, and then transferred into a different solution 5 or 30 minutes after sperm addition. For 5 minute transfer assays, sperm were minced in DVF/3. Eggs and embryos were incubated in petri dishes coated with 1% agarose dissolved in a DVF/3 solution, and plastic transfer pipettes were used to move inseminated eggs between treatments.

### Assays for the penetration of the vitelline envelope

In order to assess whether sperm penetrated the vitelline envelope of BAPTA-treated eggs, eggs were inseminated in 0 or 3 mM BAPTA with 20 μg/ml Hoechst. 20 minutes after insemination, egg jelly was removed with 45 mM β-mercaptoethanol in basic DVF/3 (pH 8.5), washed twice with acidified DVF/3 (pH 6.5), then transferred into the starting solution, DVF/3 with 0 or 3 mM BAPTA [14]. Dejellied eggs/embryos were imaged on a Leica M65FC stereomicroscope using a 10X objective, a Leica DFC450 C camera, and the Leica Application Suite software (Leica Microsystems, Switzerland). Hoechst was imaged with 358 nm excitation and 461 nm emission light, and bright-field images were obtained for the same portion of the egg. Penetration of the vitelline envelope was assessed based on whether or not sperm were visible within or beneath the vitelline envelope within 90 minutes of insemination. Images were overlaid in Photoshop CS6 (Adobe, San Jose, CA).

### Ca^2+^ assays

To estimate the readily available Ca^2+^ content within the jelly coats surrounding *X*. *laevis* eggs, freshly ovulated eggs were incubated in DVF/3, and then this DVF/3 was assayed for enriched Ca^2+^ content. Accordingly, approximately 70–150 eggs were incubated in 4 or 5 ml of DVF/3 for each trial. The DVF/3 was collected and changed every 10–30 minutes for three hours to collect and deplete readily available Ca^2+^ content of the jelly. The Ca^2+^ content of each 4 or 5 ml DVF/3 application was quantified with fura-2 photometry.

Fura-2 pentasodium salt (Alfa Aeser) was dispensed from a 100 μM stock in water for a final concentration of 100 nM in each DVF/3 aliquot. Fluorescence intensity measurements were recorded in a 1 mm quartz cuvette, in a Fluorolog 3 spectrophotometer with FluorEssence software (both from HORIBA, Jobin Yvon). Fura-2 samples were excited with 250–450 nm light, and emission was recorded at 510 nm with 3 nm slit widths. The raw photometric signals were corrected for by subtracting the fura-2 free background, collected prior to each series of measurements. The ratios were measured with 343 and 370 nm excitation light, representing the acquired excitation peaks of the Ca^2+^ bound (343 nm) and Ca^2+^ free (370 nm) fura-2 spectra [15]. The ratio of the corrected signals was calibrated [16] with equation 2:

$$[{Ca}^{2+}]=K^{*}\times \frac{\left(R-R_{min}\right)}{\left(R_{max}-R\right)}$$

where the constants R_min_ (2.4), R_max_ (6.8), and K* (28 μM), obtained from DVF/3 solutions supplemented with known amounts of CaCl_2_ ranging from 10 nM– 30 mM. The sum of total Ca^2+^ content from all washes made from a single trial was calculated and is reported as the average ± SEM.

To determine the concentration of diffusible Ca^2+^ in the jelly coat surrounding the eggs, the total Ca^2+^ content measured by fura-2 photometry was divided by the volume of solution in each wash, the number of eggs per incubation, and the average volume of the jelly coat. Jelly coat volumes were quantified from images acquired with a 10X objective on an Edmund Optics stereomicroscope, which was fitted with a pixiLINK digital camera and the μScope Essential x64 software (pixiLINK, Canada). Measurements were made from these acquired images by determining the diameter of the eggs and their surrounding jelly coat in Adobe Illustrator (San Jose, CA).

## Results

### *X*. *laevis* eggs inseminated in the absence of added divalent cations developed normally

**Fig 1:**
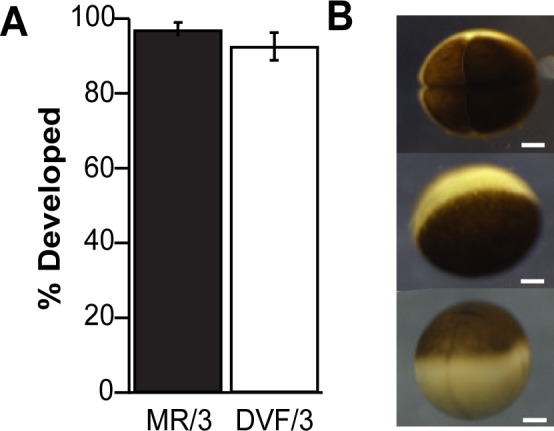
*X*. *laevis* embryos developed normally in the absence of added Ca^2+^. (A) Averaged percentage of embryos that developed cleavage furrows from eggs inseminated in MR/3 or DVF/3 (N = 153–160 eggs in 5 experimental trials). (B) Representative images of a developed *X*. *laevis* embryo at the 4-cell stage (top), an undivided egg (middle), and an embryo with faint cleavage furrows (bottom); scale bar = 250 μm.

To examine the requirement of extracellular Ca^2+^ during *X*. *laevis* fertilization and early embryonic development, we inseminated eggs in solutions with and without the addition of the divalent cations Ca^2+^ and Mg^2+^. The incidence of cleavage furrow development (N = 153–160 eggs in 5 experimental trials) was not significantly different between these two treatments with 97 ± 1% development in MR/3, compared to 93 ± 4% in DVF/3 (*X*^2^ (1) = 1.96, *P* = 0.07, Pearson’s chi-squared test) (Fig 1A). Development occurred in both experimental conditions, indicating that either divalent cations are unnecessary or that trace levels of these cations present in DVF/3 solution or the jelly surrounding the egg are sufficient to promote fertilization. Fig 1B shows representative images of embryos examined in this assay. Embryos with normal cleavage furrows were scored as developed (*top*), and eggs that did not develop cleavage furrows were scored as undivided (*middle*).

### *X*. *laevis* eggs inseminated in the presence of Ca^2+^ chelators did not develop

**Fig 2:**
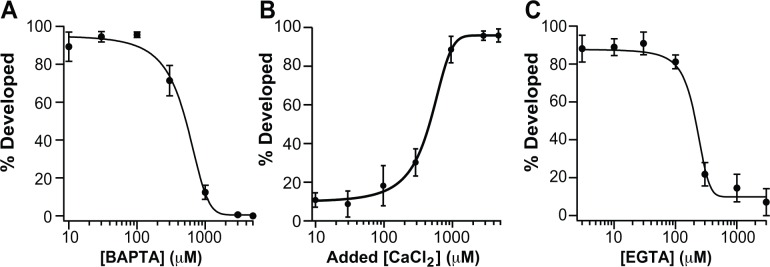
Extracellular Ca^2+^ is required for early embryonic development in *X*. *laevis*. Plots of averaged percentage of embryos that developed from eggs inseminated in DVF/3 with increasing chelator or CaCl_2_ concentrations. Each plot was fit with a sigmoidal function. (A) BAPTA concentrations ranged from 10 μM—5 mM (N = 71–190 eggs in 3–5 experimental trials). (B) Varying concentrations of added CaCl_2_ ranging from 10 μM—5 mM, with 1 mM BAPTA (N = 74–102 in 3–5 experimental trials). (C) Various EGTA concentrations ranging from 3 μM—3 mM (N = 80–167 in 4–6 experimental trials).

To investigate the absolute requirement of extracellular Ca^2+^ during fertilization and early embryonic development, we inseminated *X*. *laevis* eggs in DVF/3 that included varying concentrations of the Ca^2+^ chelator BAPTA ranging from 10 μM to 5 mM. We found that only 12 ± 4% of eggs that were inseminated in 1 mM BAPTA exhibited embryonic development (N = 190 eggs in 5 experimental trials), and that no normal development occurred in eggs inseminated in 3 mM or 5 mM BAPTA (N = 71–179 eggs in 3–5 experimental trials) (Fig 2A). By contrast, 94 ± 2% of embryos developed normally following insemination in DVF/3 (N = 237 eggs in 8 experimental trials). To measure the concentration-response relationship of BAPTA on embryonic development, we plotted the incidence of cleavage furrow development against BAPTA concentration (Fig 2A). Fitting these plots with a sigmoidal function (equation 1) yielded an average half-maximal inhibitory concentration (IC_50_) of 519 ± 76 μM (N = 71–190 eggs in 3–5 experimental trials).

To verify that the lack of embryonic development seen in eggs inseminated in BAPTA was due to the absence of extracellular Ca^2+^ rather than a non-specific effect or lack of Mg^2+^, eggs were inseminated in the presence of 1 mM BAPTA with varying concentrations of added CaCl_2_ ranging from 10 μM to 5 mM. The averaged percentage of development was plotted against the total added CaCl_2_ concentration (Fig 2B). We again used the sigmoidal function to fit these data, yielding a half-maximal effective Ca^2+^ concentration (EC_50_) of 420 ± 40 μM (N = 74–102 eggs in 3–5 experimental trials). Together these data demonstrate that extracellular Ca^2+^ rescues the effects of BAPTA treatment.

As an additional control, we examined whether another Ca^2+^ chelator, EGTA, also precluded embryonic development. To do so, we inseminated *X*. *laevis* eggs in DVF/3 with various concentrations of EGTA ranging from 3 μM to 3 mM. Similar to BAPTA, insemination in EGTA reduced the incidence of cleavage furrow development in a concentration-dependent manner with an average IC_50_ of 178 ± 25 μM (Fig 2C, N = 80–167 eggs in 4–6 experimental trials). Together with the finding that development progresses normally in solutions lacking added Ca^2+^, these results suggest that the high BAPTA or EGTA concentrations are depleting required Ca^2+^ ions present in the extracellular matrix of one or both of the *X*. *laevis* gametes.

### Ca^2+^ is required for the earliest events of fertilization and initiation of embryonic development

**Fig 3:**
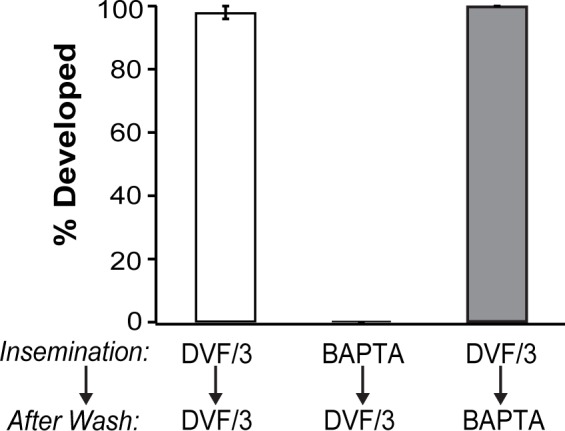
Extracellular Ca^2+^ important for the earliest events of embryonic development in *X*. *laevis*. Incidence of cleavage furrow development from eggs inseminated in DVF/3 either with 0 or 3 mM BAPTA. After 30 minutes, inseminated eggs were washed twice and moved to a new solution of DVF/3 with 0 or 3 mM BAPTA, as indicated. Embryos were assessed for the appearance of cleavage furrows 60–90 minutes after transfer (90–120 minutes after sperm addition) (N = 75–85 eggs in 4 experimental trials).

Many imperative events in embryonic development occur in the 90 minutes that it typically takes for cleavage furrows to appear. Determining which developmental events require extracellular Ca^2+^ demanded a narrower time frame. To determine when embryonic development requires Ca^2+^, we conducted transfer assays. For these experiments, eggs were inseminated in DVF/3 with or without 3 mM BAPTA. After incubating the gametes together for 30 minutes, the inseminated eggs were washed twice and transferred to a different solution: DVF/3 containing or lacking 3 mM BAPTA. Our experimental design yielded four conditions: eggs inseminated in BAPTA and transferred to BAPTA, eggs inseminated in BAPTA and transferred to DVF/3, eggs inseminated in DVF/3 and transferred to BAPTA, and eggs inseminated in DVF/3 and transferred to DVF/3. We found that none of the eggs inseminated in 3 mM BAPTA developed cleavage furrows, regardless of whether they were transferred to a 0 or 3 mM BAPTA solution (N = 75–85 eggs in 4 experimental trials) (Fig 3). These results suggest that Ca^2+^ is required during the events occurring in the first 30 minutes of embryonic development. By contrast, all embryos inseminated in the absence of BAPTA developed cleavage furrows, even after transfer to a 3 mM BAPTA solution (N = 75–85 eggs in 4 experimental trials). Interestingly, 96 ± 2% the embryos transferred from 0 to 3 mM BAPTA developed faint cleavage furrows with blastomeres that appeared less round, a phenotype not seen under typical fertilization conditions (Fig 1B *bottom vs*. *middle*).

### Sperm penetrate the jelly but not the vitelline envelope in the absence of extracellular Ca^2+^

Eggs inseminated in the presence of 3 mM BAPTA or higher failed to develop cleavage furrows in all experimental conditions examined thus far. However, these assays have not distinguished between inhibition of events occurring before or immediately after fertilization. One fertilization event thought to require extracellular Ca^2+^ is the acrosome reaction [7]. The acrosome reaction facilitates sperm penetration through the matrix surrounding the egg [17]; and in *X*. *laevis*, this particular matrix is called the vitelline envelope. Following fertilization, the egg releases its cortical granules into the perivitelline space, and enzymes contained within these granules transform the vitelline envelope into the fertilization envelope [18]. Whereas sperm readily traverse the vitelline envelope, they are unable to penetrate the fertilization envelope [19], consequently formation of this structure constitutes the slow block to polyspermy.

**Fig 4:**
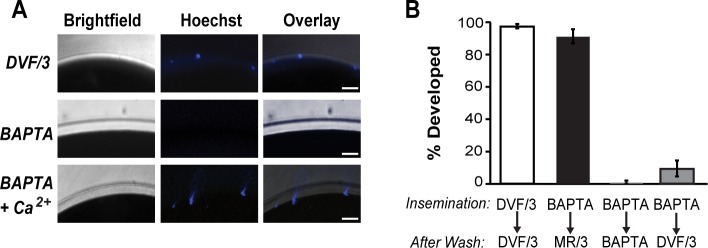
Sperm penetrate jelly but not the vitelline envelope of *X*. *laevis* eggs inseminated in BAPTA. (A) Inseminated eggs were incubated in 0 or 3 mM BAPTA, and with 0 or 3 mM CaCl_2_, were stained with Hoechst to visualize the sperm. 20 minutes following insemination, eggs were dejellied and imaged using fluorescence and bright-field microscopy to assess sperm penetration of the vitelline envelope. Representative images document the presence of Hoechst-stained sperm within the vitelline envelope of eggs inseminated in DVF/3 alone (*top*) or DVF/3 with 3 mM BAPTA and 3 mM CaCl_2_ (*bottom*) (N = 33–56 eggs in 4 experimental trials). By contrast, no Hoechst-stained sperm were evident within the vitelline envelope of eggs inseminated in DVF/3 with 3 mM BAPTA (*middle*). Scale bars represent 25 μm. Red, dashed line on overlay indicates location of envelope. (B) Incidence of cleavage furrow development of eggs inseminated in DVF/3 with 0 or 3 mM BAPTA, washed after five minutes, and transferred to a final solution as indicated (N = 75–87 eggs in 3 experimental trials).

We reasoned that if high BAPTA concentrations prevented the acrosome reaction under our experimental conditions, then sperm would only penetrate the vitelline envelope of eggs inseminated without the Ca^2+^ chelator. To test this experimentally, we imaged the perivitelline space to assay for the presence of Hoechst-stained sperm, in eggs and embryos inseminated with or without 3 mM BAPTA. To facilitate imaging, the jelly surrounding these eggs was removed using a reducing solution, which leaves the vitelline and fertilization envelopes intact [18]. The Hoechst-stained sperm were visualized with fluorescence, and bright-field microscopy was used to locate the vitelline or fertilization envelope. In four separate experiments, multiple Hoechst-labeled sperm were identified as having penetrated the vitelline envelope of all eggs inseminated in DVF/3 (N = 47 inseminated eggs in 4 experimental trials) (Fig 4A *top*), and in 55 out of 56 eggs inseminated in 3 mM BAPTA with 3 mM Ca^2+^ in 4 experimental trials (Fig 4A *bottom*). By contrast, we did not identify any sperm near eggs inseminated in 3 mM BAPTA (N = 33 inseminated eggs in 4 experimental trials) (Fig 4A. *middle*). These data suggest that sperm were unable to penetrate the vitelline envelope in the presence of BAPTA.

Finding that sperm did not penetrate the vitelline envelope or fertilize eggs in high concentrations of BAPTA led us to revisit how others were able to document fertilization and embryonic development under similarly high BAPTA concentrations. We hypothesized that in the presence of high BAPTA concentrations, sperm could penetrate the jelly coat but not the proximal vitelline envelope. To test this, we inseminated eggs in the presence or absence of 3 mM BAPTA. Five minutes after sperm addition these eggs were then washed, and transferred to solutions with various Ca^2+^ concentrations. We predicted that fertilization should be possible following the five-minute transfer as sperm should be viable and fertilization competent within 10 minutes following extraction from the testes [20]. We reasoned that if Ca^2+^ is not required for sperm to penetrate the jelly surrounding the egg, then sperm should enter the jelly coat of inseminated eggs regardless of whether or not BAPTA is present. Only sperm within the jelly coat should be transferred to the new solution. We found that embryos inseminated in 3 mM BAPTA and 5 minutes later transferred to a Ca^2+^-containing MR/3 solution, developed cleavage furrows (91 ± 4%, N = 79 in 3 experimental trials) (Fig 4B). Further supporting our hypothesis that sperm penetrate the jelly in 3 mM BAPTA but do not fertilize, we found that the first cleavage furrows appeared approximately 10 minutes later for these embryos compared to embryos inseminated in and transferred to DVF/3. As expected, we also found that embryos inseminated in, and transferred to DVF/3 developed normally (97 ± 1%, N = 87 in 3 experimental trials), and only 1 out of 75 eggs inseminated in, and transferred to 3 mM BAPTA developed (1 ± 1% in 3 experimental trials). Together, these data indicate that fertilization does not occur in 3 mM BAPTA, but that eggs inseminated in 3 mM BAPTA can be fertilized following transfer to a Ca^2+^-containing solution.

The data collected thus far indicated that at least nominal extracellular Ca^2+^ is required for fertilization. However, it was not yet clear why DVF/3, which should have a low (≤ tens of nM) Ca^2+^ content, supported fertilization and embryonic development, yet interference with embryonic development required high micromolar concentrations of BAPTA or EGTA. We hypothesized that the jelly surrounding *X*. *laevis* eggs may include a readily available store of Ca^2+^ ions, and that high BAPTA concentrations deplete this Ca^2+^ source. Another set of conditions was added to the above transfer assay to test this hypothesis. Specifically, eggs were inseminated in 3 mM BAPTA, a condition that based on our experimental observations should substantially reduce the putative Ca^2+^ store. Five minutes following sperm addition, eggs were washed in and transferred to DVF/3, a condition that should not replenish the Ca^2+^. Indeed, we found little embryonic development, 12 out of 85 eggs inseminated in 3 mM BAPTA and transferred to DVF/3 five minutes following sperm addition (10 ± 5%, in 3 experimental trials). All 12 embryos developed faint cleavage furrows (Fig 1B *bottom*). Together these results suggest that the BAPTA is removing Ca^2+^ from the extracellular matrix, and that only MR/3, and not DVF/3, replenishes this Ca^2+^ to support fertilization.

### The jelly coat surrounding the egg is enriched with freely-diffusing Ca^2+^

We next explored the hypothesis that the jelly coat surrounding the egg is enriched with readily available Ca^2+^. To do so, we estimated the diffusible Ca^2+^ content from the jelly using fura-2 photometry of DVF/3 following serial incubations of *X*. *laevis* eggs. In four experimental trials, an average of 17.5 ± 4.7 nmol of Ca^2+^ diffused from each jellied egg into the surrounding DVF/3 (N = 72–141 eggs per trial from 4 different frogs). Based on an averaged diameter of jellied eggs of 2.0 ± 0.2 mm, and an averaged diameter of 1.4 ± 0.02 mm for the egg alone, and the assumption that the egg and surrounding jelly coat are spherical, we estimate that the averaged jelly volume to be 2.8 μl (N = 11 jellied eggs from 4 frogs). Accordingly, we estimate that the averaged concentration of freely-diffusing Ca^2+^ in the jelly coat is 6.3 ± 1.7 mM.

## Discussion

### Ca^2+^ contained in the jelly coat is necessary for fertilization

Contrary to the widely held belief that extracellular Ca^2+^ is unessential for fertilization and embryonic development in *X*. *laevis* [10–13], here we present several pieces of evidence documenting that it is absolutely required. Primarily, we report that both BAPTA and EGTA effectively prevented fertilization and embryonic development in *X*. *laevis*. BAPTA and EGTA are both chelators that share a high selectivity (>10^5^) for Ca^2+^ over Mg^2+^, and each has empirically been shown to bind only a single Ca^2+^ ion [21]. The major difference between these chelators is that BAPTA is faster and less affected by pH compared with EGTA [21]. Notably, our experimental conditions lacked both Ca^2+^ and Mg^2+^. However, the ability of Ca^2+^ to recover fertilization in high BAPTA concentrations (Fig 2C) indicates that the lack of Ca^2+^ accounts for the absence of fertilization.

Given that fertilization and embryonic development occur normally in solutions lacking added Ca^2+^ or Ca^2+^-chelators, we hypothesized that the jelly coat surrounding the egg includes a store of readily diffusible Ca^2+^ that can serve as an effective Ca^2+^ buffer. This readily available Ca^2+^ store in the jelly coat would allow sperm to undergo the acrosome reaction even in conditions where the concentration of extracellular Ca^2+^ is limited. Consistent with this hypothesis, we found embryonic development to occur in eggs inseminated in 3 mM BAPTA, then washed twice and transferred to a Ca^2+^-containing MR/3 solution, but not from eggs transferred to a solution with no added Ca^2+^ (Fig 4B).

Our hypothesis that the jelly coat serves as a Ca^2+^ store is also consistent with the relatively high concentrations of chelators required to interfere with fertilization. For example, the dissociation constant (Kd) for the BAPTA-Ca^2+^ interaction at pH 7.8 is 110 nM [21], whereas here we report that the IC_50_ for the BAPTA inhibition of embryonic development is three orders of magnitude higher at 519 ± 76 μM (Fig 2A). Similarly, the Kd for the EGTA-Ca^2+^ interaction at pH 7.8 is approximately 10 nM, and our IC_50_ for the EGTA interference with embryonic development was 178 ± 25 μM (Fig 2C). Here we report that the jelly coat includes an average of 6.3 ± 1.7 mM diffusible Ca^2+^. Based on this measurement, we estimate that the half maximal Ca^2+^ concentration required to support fertilization in our experimental conditions of DVF/3, pH 7.8, supplemented with 178 μM EGTA, is approximately 6.1 mM [22]. Notably this half maximal Ca^2+^ concentration was calculated using 6.3 mM total Ca^2+^ and does not account for any diffusion before fertilization. There are likely additional fertilization-relevant conditions of the microenvironment in the jelly, such as ionic strength, also missing from this calculation. Furthermore, this value was calculated using a total Ca^2+^ concentration value of 6.3 mM; however, our measurement of 6.3 mM represents only diffusible content. Whereas the total Ca^2+^ content of the *X*. *laevis* jelly coat has not yet been measured, the ionic composition of the jelly coat for the beaked toad *Bufo arenarum* has been quantified. Using atomic absorption spectrophotometry and flame photometry, the jelly surrounding the eggs of *B*. *arenarum* reportedly includes 6.3 ± 0.9 mM Ca^2+^ [23]. Regardless, our data demonstrates that the jelly coat surrounding *X*. *laevis* eggs includes a similarly high Ca^2+^ content which is essential for fertilization.

### Ca^2+^ chelation prevents fertilization but not penetration of the jelly coat

The period in which gametes are incubated with the Ca^2+^ chelators represents an important difference between our experiments and previously published reports, thereby suggesting that Ca^2+^ is not required for fertilization; we believe this difference may account for the discrepancy between our experimental interpretations. For example, Wilkinson *et al*. reported that *X*. *laevis* eggs inseminated in 5 mM BAPTA with no CaCl_2_ or MgCl_2_, progressed with normal embryonic development [9]. For these experiments, the eggs and sperm were incubated together in 5 mM BAPTA for only five minutes before transfer to a BAPTA-free, Ca^2+^-containing solution [9]. Based on our experimental findings, we believe that fertilization did not occur until transfer to the Ca^2+^-containing solution. Here we report that sperm penetrate the jelly coat during a five-minute incubation in high BAPTA concentrations, but do not fertilize until after transfer to BAPTA-free Ca^2+^-containing solution. Moreover, we document that eggs inseminated in 3 mM BAPTA, then washed and transferred to a Ca^2+^-containing solution 5 minutes later, were fertilized and developed cleavage furrows approximately 10 minutes after the appearance of cleavage furrows under control conditions.

### Sperm require extracellular Ca^2+^ for fertilization

We hypothesize that sperm penetration of the vitelline envelope requires extracellular Ca^2+^. In our imaging experiments, we failed to locate Hoechst-stained sperm at or beneath the vitelline envelope of eggs inseminated in BAPTA. By contrast, several sperm were identified at or beneath the fertilization envelope of embryos inseminated in the absence of BAPTA, or with 3 mM BAPTA and 3 mM CaCl_2_. These experiments relied on the overlay of fluorescent images of Hoechst-stained sperm with bright-field images of the vitelline or fertilization envelopes. It is theoretically possible that the imaged sperm may not have penetrated the vitelline envelope but were instead bound to the fertilization envelope. We believe that this is unlikely primarily because sperm are unable to bind to or penetrate the fertilization envelope [19]. Additionally, the reducing conditions used to remove the jelly coat from these eggs prior to imaging should have displaced all sperm in the jelly coat.

The inability of sperm to penetrate the vitelline membrane of eggs inseminated in 3 mM BAPTA is consistent with the previously reported requirement of extracellular Ca^2+^ for physiologic processes that enable sperm to fertilize an egg. In *X*. *laevis* these processes include: undergoing the acrosome reaction [24], a process that is required for sperm to enzymatically move through the eggs’ envelope [7], and maintaining robust motility, an action that is required for sperm to traverse the thick jelly coat [8]. Extracellular Ca^2+^ is needed for sperm to undergo the acrosome reaction in various species ranging from echinoids [25] to mammals [24, 26]. In *X*. *laevis*, it has been shown that pars recta extract, which includes the physiologic ligand for the acrosome reaction, fails to evoke the acrosome reaction of sperm incubated in 50 μM of the Ca^2+^ chelators EDTA or EGTA [7]. Removal of extracellular Ca^2+^ with EGTA has additionally been shown to diminish sperm motility, an effect that is overcome with the addition of diffusible egg jelly components [8].

### Other proteins and ions may play a required role in *X*. *laevis* fertilization

While various roles for extracellular Ca^2+^ during fertilization are known, as mentioned above, the sperm and egg both undergo several other processes within the timeframe of fertilization that may also require extracellular Ca^2+^. For example, the egg envelope protein dicalcin is an S100-like Ca^2+^ binding protein that requires Ca^2+^ to function [27]. Dicalcin is thought to bind egg envelope proteins, analogous to the zona pellucida proteins found in mammals, and coordinate the vitelline envelope meshwork to mediate fertilization competence [27]. Without Ca^2+^ in the extracellular solution, it is possible that dicalcin cannot interact with vitelline envelope proteins properly to promote fertilization.

Extracellular Ca^2+^ may also be required for signaling events in the sperm. Two possible targets for Ca^2+^ in sperm include the Ca^2+^sensing receptor (CaSR), and the sperm-specific Ca^2+^ channel CatSper. The CaSR has been hypothesized to facilitate the HCO_3_^-^ signaled capacitation in mammalian sperm [28]. Capacitation refers to the required signaling events that occur in mammalian sperm between mating and fertilization in mammals [29]. A role for the CaSR in capacitation is supported by three lines of experimentation: First, CaSR expression has been documented in sperm from both rat [30] and stallion [31]. Second, calcimimetic activation of CaSR is associated with various HCO_3_^-^ signaled capacitation events including increased sperm motility and tyrosine phosphorylation [30, 31]. Third, HCO_3_^-^ signaled capacitation requires extracellular Ca^2+^ [28]. Similarly, extracellular Ca^2+^ may also be required for entry into the sperm-specific channel CatSper. In mammals, Ca^2+^ entry via CatSper is required for a swimming pattern termed hyperactivation [32], an essential behavior for sperm passage through the vestments surrounding the egg [33]. CatSper is an evolutionarily conserved sperm ion channel that also plays a crucial role in sea urchin fertilization [34]. To date, the abundance of CaSR and CatSper, and their possible role in fertilization, has yet to be determined. However, activation of either or both of these proteins may play a similar role in the preparation of *X*. *laevis* sperm for fertilization as it does for mammalian sperm.

Although BAPTA and EGTA are widely recognized as Ca^2+^ chelators, they also bind to other transition metals including Zn^2+^ [35] and Fe^3+^ [36]. It is possible that BAPTA and EGTA chelation of a metal other than Ca^2+^ is also disrupting fertilization and early embryonic development in *X*. *laevis*. Notably, Zn^2+^ has a documented requirement in mouse oocyte maturation into fertilization-competent eggs [37]. Large quantities of Zn^2+^ are released from the embryo into the extracellular space in the same timeframe of the cortical granule reactions in mammalian eggs [38]. The role that Zn^2+^ plays in *X*. *laevis* fertilization is not yet understood but it may prove to be essential. Transition metals other than Ca^2+^ are known to bind proteins required for fertilization, such as the sperm protein matrix metalloproteinase-2 (MMP-2) [39]. MMP-2 is located on the inner acrosomal membrane and aids sperm passage through the vitelline envelope, mediates binding to the oolemma of *X*. *laevis* eggs, and is required for fertilization [39]. As a metalloproteinasae, MMP-2 requires metal co-factors for their catalytic activity. While we cannot rule out the possibility that a different transition metal is important for fertilization and early embryonic development in *X*. *laevis*, we believe that the ability of Ca^2+^ to overcome the blockade imposed by high concentrations of BAPTA suggest that Ca^2+^ is necessary for these events (Fig 2B).

### Concluding remarks

We sought to resolve the controversial requirement of extracellular Ca^2+^ for fertilization and embryonic development in *X*. *laevis*. Here we present several pieces of data demonstrating for the first time that Ca^2+^ is absolutely required for fertilization and embryonic development in *X*. *laevis*. Furthermore, our data indicates that the jelly coat surrounding *X*. *laevis* eggs includes a store of readily available Ca^2+^ ions. Although the exact ionic composition of *X*. *laevis* jelly is yet to be determined, we believe the high Ca^2+^ content of the jelly coat may serve as a protective environment to optimize fertilization conditions in changing external environments.
